# Behavioral Responses and Expression of Nociceptin/Orphanin FQ and Its Receptor (N/OFQ-NOP System) during Experimental Tooth Movement in Rats

**DOI:** 10.1155/2021/9981732

**Published:** 2021-07-20

**Authors:** Zhengyu Liao, Hu Long, Zhiping Song, Yuwei He, Wenli Lai

**Affiliations:** ^1^The Affiliated Stomatological Hospital of Nanchang University, The Key Laboratory of Oral Biomedicine, Jiangxi Province, Department of Orthodontics, Nanchang, China; ^2^State Key Laboratory of Oral Diseases and National Clinical Research Center for Oral Diseases and Department of Orthodontics, West China Hospital of Stomatology, Sichuan University, No. 14, Section 3, Ren Min South Road, Chengdu 610041, Sichuan, China; ^3^Department of Anesthesiology, The First Affiliated Hospital of Nanchang University, Nanchang, China; ^4^Department of Orthodontics, Beiping Dental Clinic, Dazhou, Sichuan Province, China

## Abstract

**Objective:**

To determine behavioral testing after experimental tooth movement in rats and to explore the role of nociceptin/orphanin FQ and its receptor (the N/OFQ-NOP system) in pain induced by experimental tooth movement.

**Design:**

The mouth-wiping behavior of rats was assessed by studying behavioral responses after experimental tooth movement. The distribution of N/OFQ in the periodontal ligament, the trigeminal ganglion (TG), and the caudal one-third of the trigeminal subnucleus caudalis (Vc) was assessed by immunohistochemistry. The variations in N/OFQ expression in the TG and Vc were measured by Western blotting. The ongoing changes in the gene expression of the prepronociceptin gene and opioid receptor-like 1 receptor were assessed in the TG and Vc by real-time polymerase chain reaction (RT-PCR).

**Results:**

Overall, the mouth-wiping behavior increased significantly. The behavior first increased and then gradually decreased to a low level, showing cyclical variation. N/OFQ immunoreactivity increased in the periodontal ligament after tooth movement. ppN/OFQ mRNA and protein levels showed a time-dependent increase in the TG and were positively correlated with pain stimulus. NOP gene levels showed large fluctuations. In the Vc, the expression and changes in the N/OFQ-NOP system showed the opposite trend as those noted in TG and the periodontal membrane.

**Conclusion:**

The N/OFQ system may have a complex regulatory function in the pain induced by tooth movement and may be related to inflammation caused by orthodontic tooth movement and periodontal damage. The specific mechanism remains to be further studied.

## 1. Introduction

Orthodontic treatment is based upon the application of an orthodontic force to correct the dislocation of teeth, dental arch, or jaws. During orthodontic treatment, especially during the initial stages of treatment, such as insertion of separators and arch wires, the most troublesome problems patients typically encounter clinically are pain and discomfort [1, 2]. Some patients may even stop orthodontic treatment as a result of the unpleasant emotional experience of pain [3, 4]. Therefore, the mechanisms of orthodontic pain and methods for pain alleviation have attracted considerable attention in orthodontics.

In the past, the search for the mechanisms of orthodontic pain mainly focused on neurotransmitters (and their receptors) and immune cell products [4–8]. It has been demonstrated that nociceptin/orphanin FQ (N/OFQ) and its nociceptin/orphanin peptide receptor (NOP) belong to the endogenous opioid system and play an important role in pain control [9]. Similar to classical opioid peptide members, the N/OFQ system is widely distributed in the central nervous system and in many tissues and organs. However, the N/OFQ system is very different from the classical opioid system in terms of biological functions, especially pain regulation [10–12]. Most studies have suggested that the N/OFQ-NOP system can modulate pain in a bidirectional manner, which depends on several factors, such as the regulatory level in the nervous system (peripheral, spinal, or supraspinal) and the characteristics of pain (inflammatory pain, acute pain or neuropathic pain) [13–17]. However, to date, no consensus on the role of the N/OFQ-NOP system in pain control has been reached. Previous studies on orthodontic pain were usually limited to one specific regulating level, which showed a certain one-sidedness. Although similar studies have revealed N/OFQ-NOP expression changes at the trigeminal nucleus level after experimental tooth movement in rats, the associations of these expression patterns with orthodontic pain have not been elucidated [18–20].

Therefore, in this study, we explored the role of N/OFQ in orthodontic pain by assessing orofacial pain and examining the expression of N/OFQ separately in the trigeminal nucleus, trigeminal ganglia, and periodontal tissues of rats during tooth movement.

## 2. Materials and Methods

### 2.1. Animals

In this experiment, male Sprague Dawley rats weighing 200∼300 g were used according to the guidelines provided by the National Institute of Health (NIH), USA. Rats with abnormal appearance, action, and body temperature were excluded. Rats were kept in standard clean plastic cages with free access to water and granulating feed at room temperature at 24–27°C with a 12-hour light-dark cycle for at least 5 days prior to the experiments. The study was approved by the ethical board of the State Key Laboratory of Oral Diseases, Sichuan University and guided by the International Association of Pain Research [21].

After anesthetization via intraperitoneal injection of 1% pentobarbital sodium (145 mg/kg), the rats were ligated with a NiTi alloy closed spring appliance (0.012 in × 9 mm, Shinwa, China) between the left maxillary first molars and incisors to induce mesial movement of the left maxillary first molars. When the force was applied, the spring was stretched. According to the results of previous studies, we chose 40 g force as the appropriate experimental force. The force (40 g) produced by the spring was determined using a pulling force tensiometer (Tiantian, Changsha, China) [22], and the force device (NiTi spring) was placed in the mouth for 4 hours, 1 day, 3 days, 5 days, 7 days, and 14 days. The sham-treated rats (the control group) received the same operation with no force produced by the springs (a force device (NiTi) spring was placed in the mouth, and no force was applied). The mouths were examined every day to assess whether the spring fell off. Rats in which the spring fell off prematurely were excluded, and two animals were excluded due to the relocated devices. Detailed grouping information is depicted in Table 1.

After anesthetization via intraperitoneal injection of 1% pentobarbital sodium (145 mg/kg), rats were sacrificed via perfusion with 4% cold paraformaldehyde in phosphate buffered saline (PBS) on the 1st day, 3rd day, 5th day, 7th day, and 14th day after tooth movement (6 rats for each group at each time point, perfusion only for animals used for Immunochemistry staining studies and not for Western blot analysis and real-time PCR). Additionally, 6 rats not subjected to any intervention were sacrificed to obtain baseline data. The left maxillary molars, alveolar bones, left trigeminal ganglia, and trigeminal nucleus of the medulla oblongata were removed and stored in liquid nitrogen.

### 2.2. Behavior Testing

Behavior testing for orofacial pain was conducted on the 1st day, 3rd day, 5th day, 7th day, and 14th day after tooth movement (6 rats for each group at each time point). The rats in the 4 h group did not undergo behavior testing due to potential anesthesia interference. Additionally, 6 rats were tested without any intervention as baseline data. Rats were monitored from 7 pm to 9 pm with a background noise of 45 dB in a lucid glass cage (30 cm × 30 cm × 30 cm) with a thin layer of wood bits and a mirror at the back. After being left in the cage for 15 min, each rat was videotaped for 10 min three times [23], and we averaged the three measurements. A tester blinded to the grouping information of rats recorded the time each rat spent on mouth wiping.

### 2.3. Immunochemistry Staining

After decalcification of teeth and bones for 45 days, the tissues were cut serially at a thickness of 5 *μ*m, and 8 sections were randomly selected for immunohistochemical staining. These sections were originally incubated with rabbit anti-N/OFQ polyclonal antiserum (1 : 100, Santa Cruz, USA) for 3.5 h at 37°C, incubated with goat anti-rabbit IgG antibody (1 : 100, Zhong Shan Biotech), and finally developed with a DAB Horseradish Peroxidase Color Development Kit.

### 2.4. Western Blot Analysis

After determination of the total protein from trigeminal ganglia and the medulla oblongata tissue, lysates were fractionated by 15% SDS PAGE, transferred onto nitrocellulose (NC) membrane and then incubated with primary rabbit polyclonal antibody against ppN/OFQ (1 : 500, Santa Cruz) and secondary antibody (1 : 5000, Zhongshan Biotech). *ð*-Actin (Zhong Shan Biotech) was used as a reference protein. The band intensity was estimated with Image Quant 5.2 software, and the protein level of ppN/OFQ in the same sample was normalized to that of *ð*-actin.

### 2.5. Real-Time PCR

Primers and probes were designed according to the PNOC gene, NOP gene, and GAPDH housekeeper gene sequences in the NCBI GenBank. RNA was extracted with TRIzol reagent (Gibco BRL) following the operation manual. First-strand cDNA was synthesized from 1 *μ*g total RNA in a 20 *μ*l volume using random hexamers as primers. A 20 *μ*l portion of the resultant cDNA was amplified in a final volume of 50 *μ*l employing Premix Ex Taq (Takara) as the thermostable enzyme. Specific primers for the rat PNOC (prepronociceptin) and NOP receptors were as follows: PNOC (122 bp), forward primer: 5′- TGCTCTCCAGCGTGTTCAG -3'; reverse primer: 5′- GAAGACCTTCTCTTCACACT -3'; TaqMan fluorescence probe: 5′- CTGCCTCACCTGCCAGGAG -3'; NOP (167 bp) forward primer: 5′- GTGTTCCTGTTGCCATCAT -3'; reverse primer: 5′- GAGGCTGTAGCAGACAGAGAT -3'; TaqMan fluorescence probe: 5′- CGAGTGCCTGGTGGAGATCC -3'. RT-PCR was performed employing the Takara SYBR PrimeScript RT-PCR kit (v Biotechnology, Dalian, China) on an FTC-3000 qPCR System (Funglyn Biotech Inc, Canada). The following amplification curve was applied: 120 s at 94°C followed by 35 cycles of 20 s at 94°C, 30 s at 55°C, 30 s at 60°C and a final extension step of 7 min at 60°C. PCR without the RT step was included in all experiments (data not shown). Ten microliters of each PCR product was run on a 1.0% agarose gel in 0.53 Tris-borate EDTA buffer, dyed with ethidium bromide (0.5 *μ*g/ml), and then photographed on a UV box using Canon 60D film.

### 2.6. Statistical Analysis

The data were expressed as the mean ± standard error of the mean (SEM). One-way ANOVA (least significant difference, LSD) and Student's *t*-test were used to analyze the difference in time spent on mouth wiping and N/OFQ expression (including the immunohistochemistry of tooth and alveolar bone as well as the results of Western blot and real-time PCR) using SPSS 18.0. A *p* value less than 0.05 was considered significant.

## 3. Results

### 3.1. The Effect of Experimental Tooth Movement on Orofacial Pain Level

**Figure 1:**
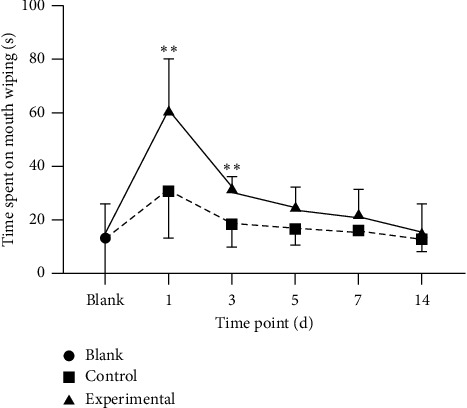
The time spent on mouth-wiping behaviors is used as a surrogate measure of the orofacial pain level. Note: compared with the blank group, the experimental group exhibited increased activity on the 1^st^ and 3^rd^ days. Bars represent the mean ± SEM. ^*∗∗*^*p* < 0.01.

As displayed in Figure 1, our results revealed that the time spent performing mouth-wiping behaviors, which served as a measure of orofacial pain level, increased significantly and peaked on the 1st day following experimental tooth movement (*p* < 0.01), decreased on the 3rd day (*p* < 0.01), and gradually returned to the baseline level in the experimental group. In contrast, orofacial pain levels did not differ from the baseline levels at all time points in the control group.

### 3.2. The Effect of Experimental Tooth Movement on N/OFQ Expression in the Periodontal Ligament

**Figure 2:**
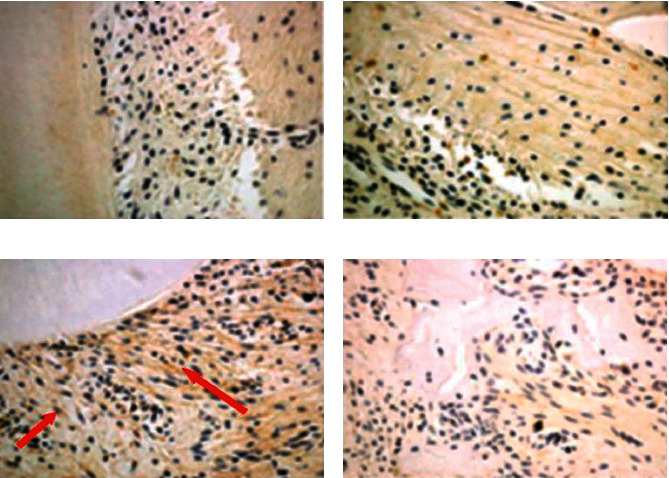
Immunohistochemical staining of the periodontal ligament (the left maxillary molar and alveolar bones). (a) Blank group (40x). (b) Experimental group, 1st day (40x). (c) Experimental group, 5th day (40x). (d) Experimental group, 14th day (40x).

**Figure 3:**
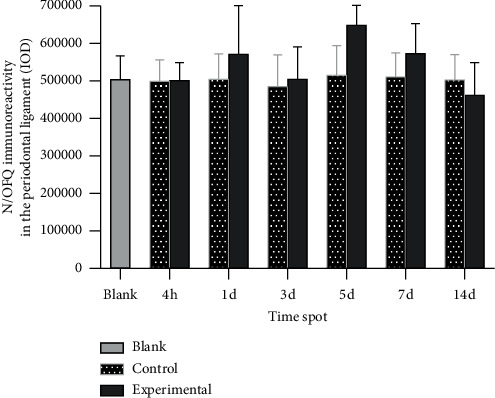
Changes in N/OFQ immunoreactivity in the periodontal ligament (IOD). Immunohistochemical image grayscale analysis of the integral optical density results. Bars represent the mean ± SEM. No significant differences were noted between the experiment group and the control group on the same day, *p* < 0.05 (compared with the control on that day).

Immunochemistry staining showed that the N/OFQ immunoreactivity significantly increased in the periodontal ligament (the compression side) on the 1st day and decreased on the 5th day after tooth movement in the experimental group (Figure 2), which is consistent with the changes in orofacial pain level. These results indicate that the N/OFQ system is closely related to the pain caused by local injury. No significant differences were noted between the experiment group and the control group on the same day. (*p* < 0.05) (and Figure 3).

### 3.3. The Effect of Experimental Tooth Movement on ppN/OFQ and NOP Expression in the Trigeminal Ganglia

**Figure 4:**
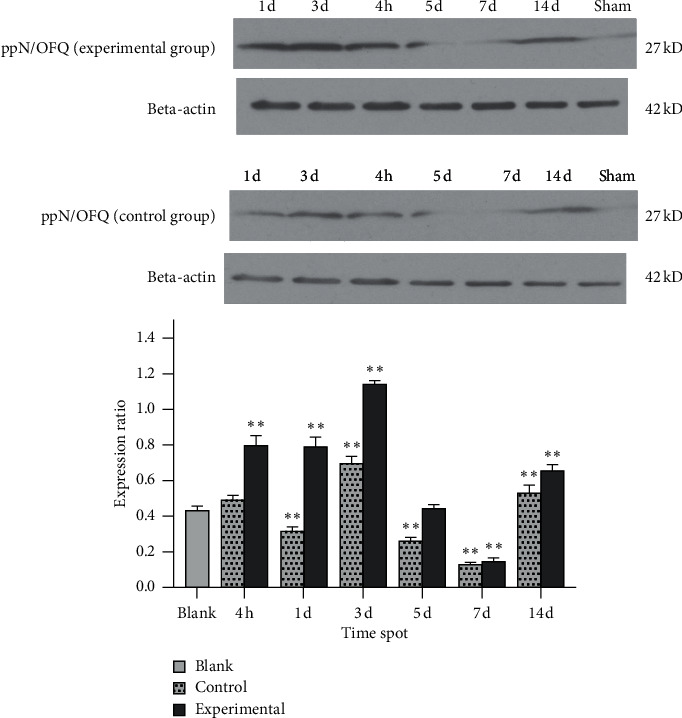
ppN/OFQ protein expression in the trigeminal ganglion tissue of rats. Note: except for the 5th day, PPN/OFQ protein expression levels at other time points in the experimental group were significantly different compared with the blank group. Except for at 4 hours, PPN/OFQ protein expression levels at other time points in the control group were significantly different compared with the blank group. Bars represent the mean ± SEM. ^*∗∗*^*p* < 0.01.

**Figure 5:**
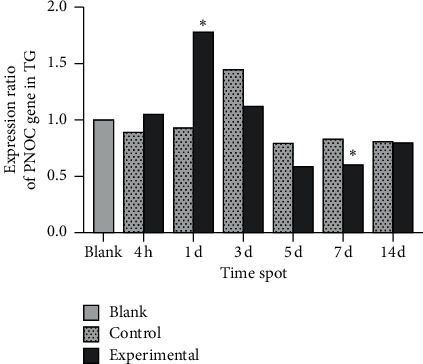
PNOC mRNA levels in the trigeminal ganglia. Note: significant differences were noted between the experimental group and the blank group on the 1^st^ and 7^th^ days. No significant difference was noted between the control group and the blank group at each time point. Data were expressed as means ± SEM. ^*∗∗*^*p* < 0.01.

**Figure 6:**
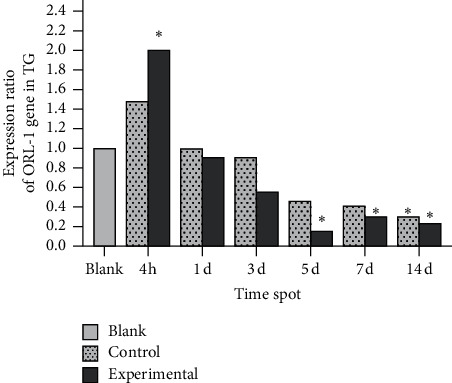
NOP mRNA levels in the trigeminal ganglia. Note: on the 5^th^, 7^th^, and 14^th^ days, the difference between the experimental group and the blank group was statistically significant. No significant difference was noted between the control group and the blank group except on the 14^th^ day. Data were expressed as means ± SEM. ^*∗*^*p* < 0.05.

In the experimental group, ppN/OFQ mRNA and protein levels in the trigeminal ganglia significantly increased 4 h after tooth movement (*p* < 0.01), which was positively correlated with pain stimuli and indicated increased sensitivity compared with that in periodontal tissues. Subsequently, ppN/OFQ mRNA and protein levels in the trigeminal ganglia peaked on the 1st and 3rd days, respectively (*p* < 0.01), followed by a gradual decline and then a notable increase on the 14th day (*p* < 0.01). We divided the relative gene copy number of each experimental group by the relative gene copy number of the blank group to obtain the gene expression ratio level of each group relative to the baseline. The changes in the control group were similar but to a lesser extent (Figures 4 and 5). The nociceptin receptor NOP gene level immediately increased at 4 h, which is consistent with ppN/OFQ expression. Then, the levels decreased rapidly and remained at a low level. The processed side of the control group showed similar changes (Figure 6). The above results suggested that the N/OFQ systems are closely related to the injury caused by tooth movement in the peripheral ganglia, playing a pain regulatory role not only in the early stage but also throughout the entire process of tooth movement, which still needs to be further assessed.

### 3.4. The Effect of Experimental Tooth Movement on ppN/OFQ and PNOC Expression in Vc

**Figure 7:**
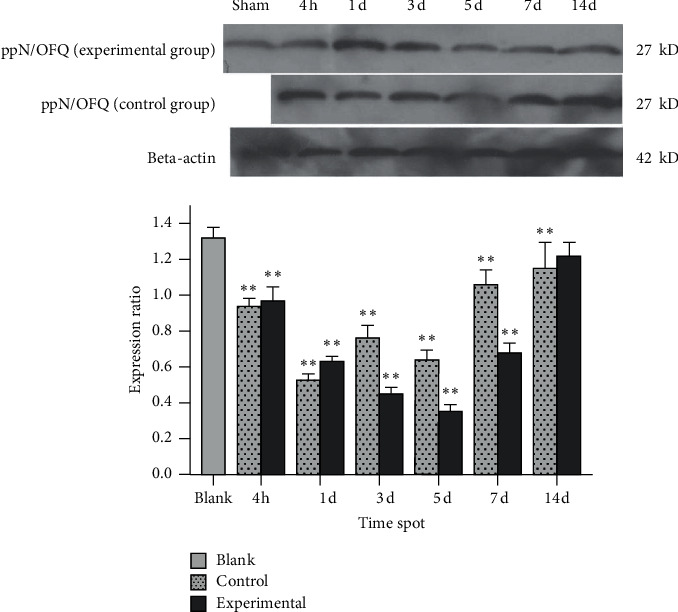
ppN/OFQ protein expression in the Vc of rats. Note: except for the experimental group on the 14^th^ day, PPN/OFQ protein expression levels at other time points in the experimental group were significantly different compared with the blank group, and PPN/OFQ protein expression levels at each time point in the control group were significantly different compared with the blank group. Bars represent the mean ± SEM. ^*∗∗*^*p* < 0.01.

**Figure 8:**
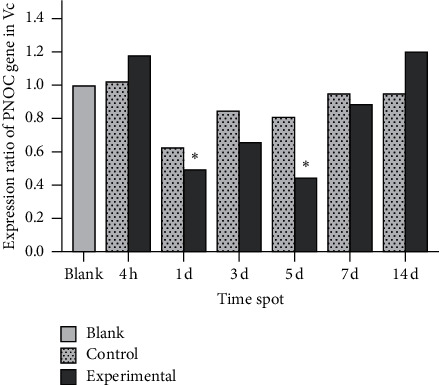
PNOC mRNA levels in the Vc. Note: significant differences were noted between the experimental group and the blank group on the 1^st^ day and the 5^th^ day, but no significant differences were noted between the control group and the blank group at each time point. Data were expressed as means ± SEM. ^*∗*^*p* < 0.05.

**Figure 9:**
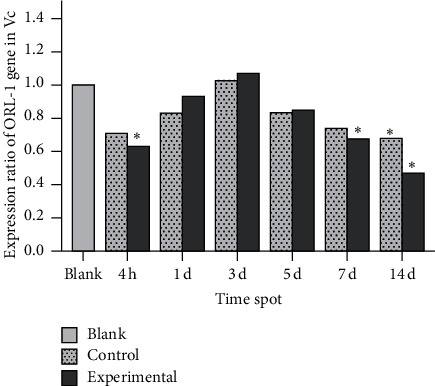
NOP mRNA levels in the Vc. Note: significant differences were noted between the experimental group and the blank group at 4 hours, 7 days, and 14 days. No significant difference was noted between the control group and the blank group except on the 14^th^ day. Data were expressed as means ± SEM. ^*∗*^*p* < 0.05.

In control rats, many N/OFQ-positive cells were noted mainly in the small and medium neurons of the Vc. After experimental tooth movement, ppN/OFQ protein and mRNA levels showed some temporally downward changes in Vc at the experimental side in negative correlation with the pain stimulus, which initially decreased, reached the lowest point on the 5th day (*p* < 0.05) and then increased (Figures 7 and 8). In contrast, mRNA levels of the nociceptin receptor NOP showed an immediate decrease at 4 h (*p* < 0.05) followed by a slow increase until the 3rd day and a second decrease (*p* < 0.05), and the processing side of the control group showed similar changes (Figure 9). The changes in the N/OFQ system in the Vc, which are inconsistent with those in the trigeminal ganglia and periodontal ligament after experimental tooth movement and periodontal injury, prompted a complex regulatory function of the N/OFQ system on pain. This process exerted a specific effect in the early stages after orthodontic tooth movement and periodontal inflammatory injury.

## 4. Discussion

Pain and discomfort are two major problems in subjects undergoing orthodontic treatment and are discerned by patients typically after bonding and/or adjustment of orthodontic appliances [24–26]. Experimental tooth movement in rats has been demonstrated to yield c-fos, a marker of neuronal activity expressed in the Vc, implying that the model of experimental tooth movement could successfully induce orofacial nociceptive stimulation [4]. Mouth-wiping behavior has been previously suggested as a reliable and reproducible assessment of pain arising from experimental tooth movement [19], which is effective when considering the mechanisms mediating orthodontic pain [27]. Our results showed that the time spent on mouth wiping in the experimental group extremely increased after tooth movement. Specifically, the time peaked on the 1st day and then gradually returned to the baseline level on the 14th day, which supported the validity of face-based measurements in this pain-inducing model. Moreover, the time-dependent changes in pain level were consistent with the clinical fact that the strongest pain responses in orthodontic therapy tend to occur immediately after inserting the initial wire into the brackets. Notably, the mouth-wiping behaviors of the control group also increased on the 1st day compared to the blank group, although without statistical significance, suggesting that periodontal tissue damage inevitably caused by the ligature passing through the interproximate space may lead to a temporary increase in mouth-wiping behavior, which is significantly reduced after the animal adapts to the pain.

N/OFQ and NOP receptors are located in the central nervous system (CNS), the peripheral nervous system, and parts of the peripheral tissues, such as the vascular wall, adrenal gland, liver, small intestine, vas deferens, ovary, spleen, and peripheral leukocytes [11]. Previous studies have shown that N/OFQ has opposite influences on at least three levels, including the primary afferent nerve fibers (nociceptors) and spinal and supraspinal nerve systems. Our study found that a limited amount of N/OFQ was positively expressed in the periodontal ligament and apical roots, which started to increase 1 day after tooth movement and gradually decreased after a week. This trend was possibly due to increased adaptation of local tissues to external stimulation. On the other hand, the spring force decayed when tooth movement achieved certain mobility, and the changes in the periodontal tissues of the experimental group were derived from stasis, which also suggested that N/OFQ expression changes with the intensity of the stimulus. However, a more accurate quantitative study of N/OFQ in periodontal tissue is needed in the future.

Our study also showed that tooth movement could cause increased N/OFQ expression in the trigeminal ganglia of the loading side. In addition, immunohistochemical staining showed that a limited number of N/OFQ-positive cells in the trigeminal ganglion of normal rats were mainly located in small- and medium-sized neurons of the myelin sheath. This finding is consistent with physiological studies indicating that the nociceptive signals from the mouth were mainly transduced by the middle and small neurons in the trigeminal ganglion, whereas the large neurons were mainly involved in the conduction of the tactile and deep sensory information [28].

Due to the low molecular weight of N/OFQ, which is difficult to detect, we used the precursor N/OFQ, prepronociceptin/orphanin (ppN/OFQ), to represent N/OFQ expression levels. In the experimental group, ppN/OFQ protein expression in the trigeminal ganglia was significantly increased after 4 hours following tooth movement, reached the highest level on the 3rd day, and then decreased. This changing trend was consistent with behavioral responses, suggesting that peripheral N/OFQ may contribute to the regulation of pain in the early stage after orthodontic tooth movement. The initial increase in ppN/OFQ expression was probably induced by the augmented consumption of N/OFQ binding to its receptor at the early stages of pain, which was alleviated as the body adapted to external stimuli. Moreover, ppN/OFQ expression levels in the TG of the control group kept changing, suggesting N/OFQ might be closely related to the inflammatory pain induced by the spring itself, and the experimental results need to be verified by further experiments. When the spring is placed in the mouth of rats as a foreign body, it may cause local damage and inflammatory response, resulting in changes in the level of orphanin in the peripheral tissues. In addition, N/OFQ expression levels were positively correlated with the stimulus strength.

The distribution of N/OFQ and its receptor NOP is consistent and has been found in the sensory neurons of the dorsal root ganglia and the trigeminal ganglion [29]. In our study, experimental tooth movement caused NOP mRNA expression levels in the trigeminal ganglion to peak after 4 hours and then gradually decreased to a low level. Low NOP expression levels are potentially due to a negative feedback effect activated by the initial increase in NOP in response to early pain after loading.

Facial pain signals are transmitted upward to the spinal cord of the trigeminal nerve and end in the nucleus of the spinal tract of the trigeminal nerve. The Vc and its adjacent regions are considered closely related to the transmission of facial injury information, which is similar to the function of the dorsal horn in the spinal cord [30, 31]. We found that ppN/OFQ protein expression in the Vc decreased 4 hours after tooth movement, and ppN/OFQ protein levels in the Vc, TG, and periodontium were not completely equivalent. These findings indicated that the ppN/OFQ in the medulla oblongata might also play an essential role in orthodontic pain transmission, but ppN/OFQ plays a different role in the trigeminal ganglia and periodontal tissues, which is consistent with a large number of studies indicating that the pain regulatory role of the N/OFQ-NOP system is different depending on the regulation level, as mentioned above [32, 33]. After reaching the lowest protein expression level on the 3rd to 5th day after orthodontic tooth movement, ppN/OFQ increased at the later stage of tooth movement, which may be related to the adaptive changes in N/OFQ regulated by the medulla.

On the other hand, NOP mRNA expression in the Vc showed the lowest level at 4 hours, gradually increased with a boost on the 3rd day, and then began to decrease, suggesting that the N/OFQ receptor displays cyclic variations over time after orthodontic tooth movement or periodontal tissue injury, which may be due to the changes in the binding rate of N/OFQ or other adjusting mechanisms of the N/OFQ system. Therefore, further studies are warranted, such as pain evaluation of animal injections with exogenous agonists and antagonists of N/OFQ and NOP receptors.

Based on the above findings, it is obvious that further investigations on the mechanism by which the N/OFQ system regulates pain are required. Our study did not involve the regulatory mechanisms of the central nervous system at levels higher than the medulla; hence, the expression changes of the N/OFQ system in advanced neural centers after experimental tooth movement remain unknown. In the future, we want to observe the changes in the expression of this system by injecting the corresponding antagonists and agonists of nociception and detecting the expression changes in this system in combination with behavioral studies after orthodontic tooth movement.

## 5. Conclusions

The N/OFQ system plays an essential role in orofacial pain caused by orthodontic tooth movement, which varies among different levels of the nervous system, such as peripheral terminals, trigeminal ganglia, and the trigeminal nucleus.

## Figures and Tables

**Table 1:** Grouping of animals.

Time Spot	0 hour	4 hours	1 day	3 days	5 days	7 days	14 days
Blank	6						
Control		6	6	6	6	6	6
Experimental		6	6	6	6	6	6

To prevent interference from anesthesia, the 4 hr group was not assessed in the behavior test.

## Data Availability

The original data used to support the findings of this study are available from the corresponding author upon request.
